# Supplementary material for: A global survey of diurnal offshore propagation of rainfall
Source: Nat Commun. 2022 Dec 2;13:7437. doi: 10.1038/s41467-022-34842-0 (PMC9718862; doi:10.1038/s41467-022-34842-0)
Supplement: Supplementary file 1 — Supplementary Information [file 41467_2022_34842_MOESM1_ESM.pdf]

Nature Communications

A global survey of diurnal offshore propagation of rainfall

Junying Fang & Yu Du

Supplementary Information

This supplement contains four additional figures and one additional table that bolster the analysis presented in the main paper.

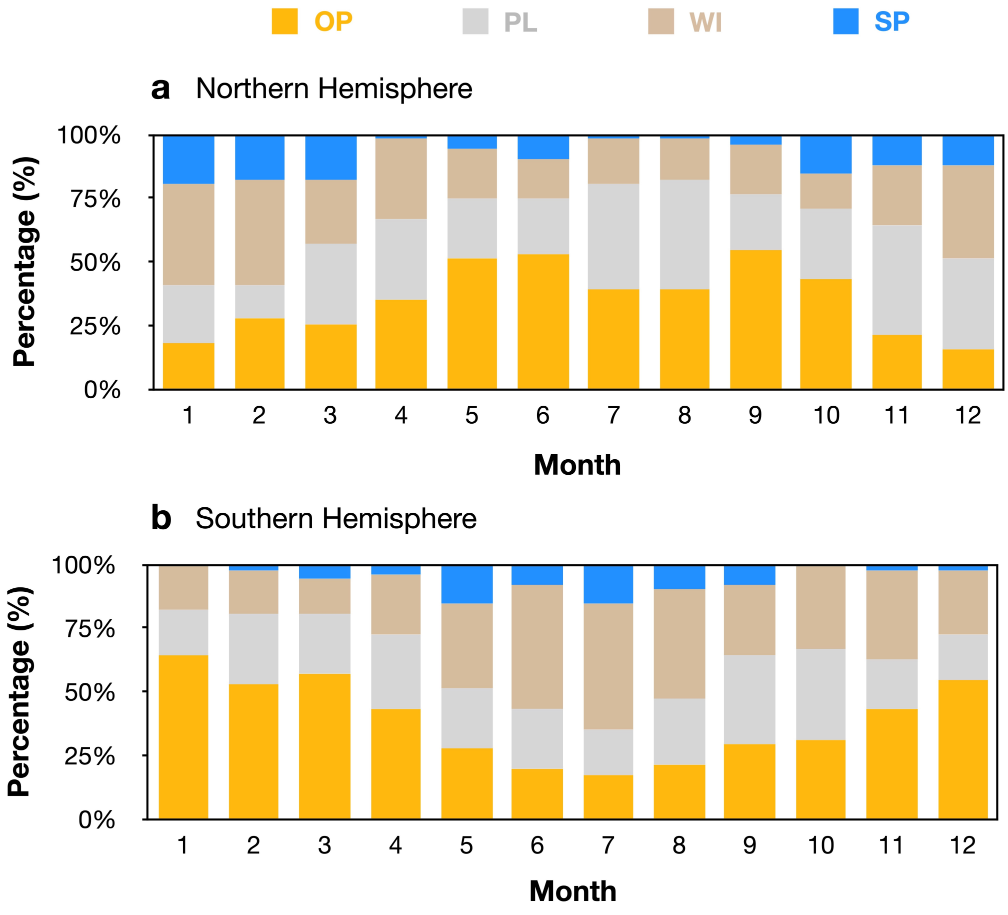

Supplementary Fig. 1: Responses of the diurnal pattern proportion to seasonal variation in each hemisphere. OP, PL, WI and SP denote offshore propagation, phase-locked, weak or incoherent and shoreward propagation diurnal patterns, respectively. Fifty-one coasts were selected in each hemisphere.

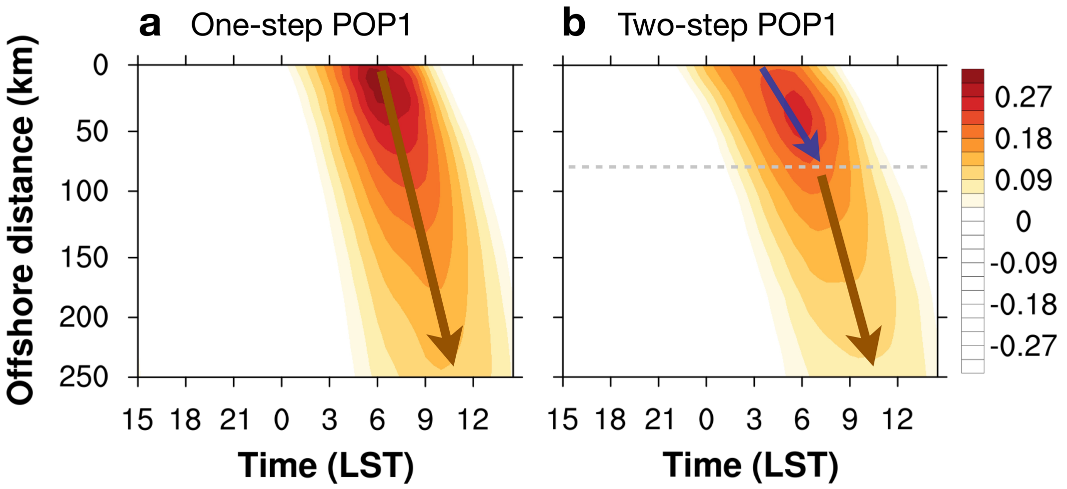

Supplementary Fig. 2: Composite distance–time Hovmöller diagrams of the hourly precipitation deviation (shaded, mm) for the one-step and two-step POP1. The two-step POP1 samples accounted for 29% of POP1 samples, 35 in total. The one-step POP1 samples accounted for 71% of POP1 samples, 90 in total. The brown and blue arrows indicate the diurnal rainfall propagation driven by IGWs and density currents (DCs), respectively. The propagation speed transition for the two-step POP1 is indicated by the grey dashed line. POP is pronounced offshore propagation with sufficient moisture ($q > 11$ g kg^{-1}), and POP1 is post-midnight POP.

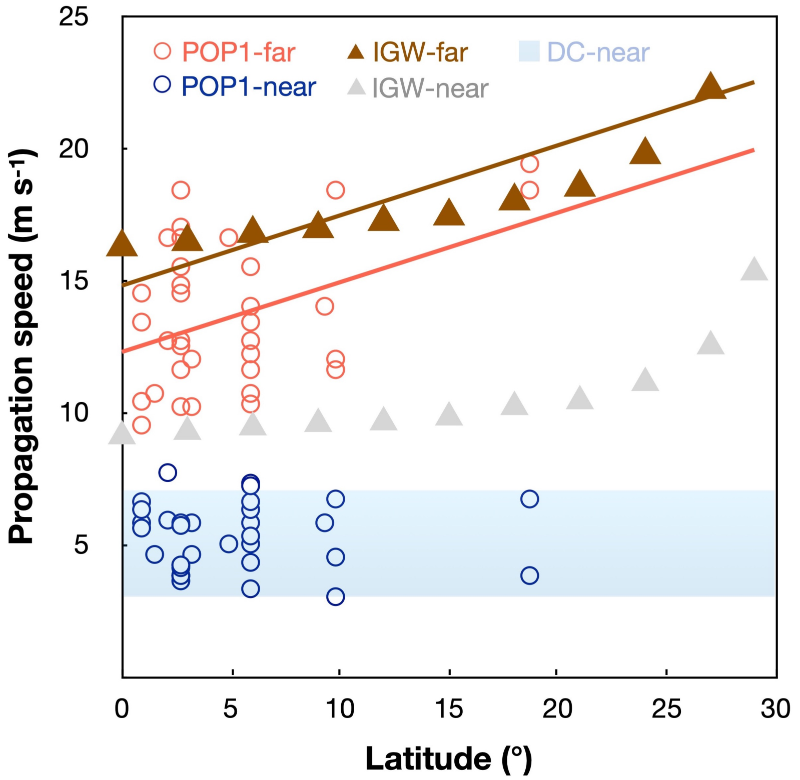

Supplementary Fig. 3: The differences of near- and far-shore propagation speed of the two-step POP1 samples. The far-shore propagation speed of the two-step POP1 samples (red circles) and analytical solutions of inertia–gravity wave forced by the terrain/land–sea thermal contrast (IGW, brown triangles), and near-shore propagation speed of the two-step POP1 samples (blue circles), IGW analytical solutions (grey triangles) and density current (DC) characteristic range (sky blue range, 3–7 m s^{-1}) with the latitude. The least square fit lines between the far-shore propagation speed and latitude for the two-step POP1 ($r = 0.41$, $P < 0.02$) and IGWs ($r = 0.83$, $P < 0.005$) are indicated by red and brown lines, respectively. POP is pronounced offshore propagation with sufficient moisture ($q > 11$ g kg^{-1}), and POP1 is post-midnight POP.

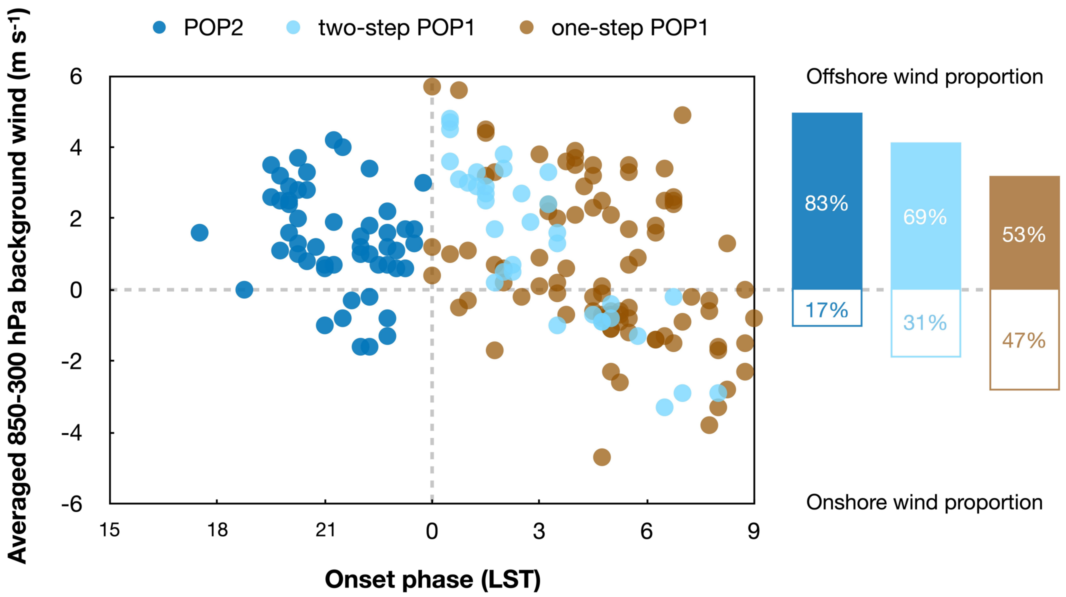

Supplementary Fig. 4: The relationship of background wind and onset phase among three types of POPs. Positive (negative) values of the averaged 850–300 hPa background wind indicate its offshore (onshore) component. The bars indicate the proportion of offshore and onshore background wind for each type. The POP samples are identified as pronounced offshore propagation with sufficient moisture ($q > 11$ g kg^{-1}). The POP1 (POP2) samples are identified as post-midnight (pre-midnight) POP samples.

Supplementary Table 1 Seasonal differences in OP features.

	Summer	Winter
Mean background wind (m s^{-1})	0	0
Mean latitude (°)	21	21
Mean specific humidity (g kg^{-1})	8.5	5.9
OP samples amount	155	61
OP percentage of samples	51%	20%
OP percentage of precipitation	73%	35%
OP mean hourly precipitation (mm)	0.21	0.15
OP mean diurnal amplitude (mm)	0.10	0.07
POP samples amount	67	19
POP mean speed (m s^{-1})	11.2	10.1
POP mean onset phase (LST)	2	3
POP mean distance (km)	635	393

Summer (winter) is defined as JJA (DJF) in the Northern Hemisphere and DJF (JJA) in the Southern Hemisphere. Positive (negative) values of the background wind indicate the offshore (onshore) component of the averaged 850–300 hPa background wind. The specific humidity takes its value at 850 hPa. OP denotes diurnal offshore propagation of rainfall. The POP samples are identified as OP samples with sufficient moisture ($q > 11$ g kg^{-1}).
